# Geographical Origin Traceability of Atractylodis Macrocephalae Rhizoma Based on Chemical Composition, Chromaticity, and Electronic Nose

**DOI:** 10.3390/molecules29214991

**Published:** 2024-10-22

**Authors:** Ruiqi Yang, Yushi Wang, Jiayu Wang, Xingyu Guo, Yuanyu Zhao, Keyao Zhu, Xintian Zhu, Huiqin Zou, Yonghong Yan

**Affiliations:** School of Chinese Materia Medica, Beijing University of Chinese Medicine, Beijing 102488, China; yangrq@bucm.edu.cn (R.Y.); 20230935206@bucm.edu.cn (Y.W.); 20230935245@bucm.edu.cn (J.W.); 20230941454@bucm.edu.cn (X.G.); 20220935197@bucm.edu.cn (Y.Z.); 20220935198@bucm.edu.cn (K.Z.); 20220935199@bucm.edu.cn (X.Z.)

**Keywords:** Atractylodis Macrocephalae Rhizoma, origin traceability, chromaticity, electronic nose, quality evaluation

## Abstract

Atractylodis Macrocephalae Rhizoma (AMR) is a traditional Chinese medicine used for gastrointestinal diseases. With increased demand, there are more and more places of cultivation for AMR. However, the quality of AMR varies from place to place, and there is no good way to distinguish AMR from different origins at present. In this paper, we determined the content of eight chemical components including 60% ethanol extracts, essential oil, polysaccharides, atractylenolides, and atractylone, obtained the color parameters of AMR powder by colorimetry, and odor information was captured by the electronic nose, all of which were combined with machine learning to establish a rapid origin traceability method. The results of the principal component analysis of the chemical components revealed that Zhejiang AMR has a high comprehensive score and overall better quality. The Kruskal–Wallis test demonstrated that there are varying degrees of differences in chemical composition and color parameters across the different origin. However, the accuracy of the classification model is low (less than 80%), making it difficult to distinguish between different origins of AMR. The electronic nose demonstrated excellent classification performance in the traceability of AMR from different origins, with accuracy reaching more than 90% (PLS-DA: 96.88%, BPNN: 96.88%, PSO-SVM: 100%). Overall, this study clarified the quality differences of AMR among different origins, and a rapid and precise method combining machine learning was developed to trace the origin of AMR.

## 1. Introduction

Atractylodis Macrocephalae Rhizoma (AMR), named “Baizhu” in Chinese, is a member of the Compositae family, which is the dried rhizome of Atractylodis Macrocephalae Koidz, with a fresh fragrance. Its functions include invigorating the spleen and invigorating qi, drying dampness and diuresis, antiperspirant, and anti-fetus. It is used to treat spleen deficiency and lack of food, abdominal distension and diarrhea, dizziness and palpitations, edema, spontaneous sweating, and fetal restlessness [1]. In addition, several previous studies have reported that AMR has a variety of pharmacological activities such as anti-tumor [2], neuroprotection [3], immunomodulatory [4], improving glucose and lipid metabolism and insulin resistance, regulating gut microbiota [5,6], anti-inflammatory, etc. [7]. Most of the studies have shown that pharmacologically active components of AMR are focused on polysaccharides, essential oil and sesquiterpenes, particularly atractylenolide I (AE I), atractylenolide II (AE II), atractylenolide III (AE III), and atractylone (AO).

Due to the remarkable therapeutic effects of AMR, market demand is constantly increasing. With the depletion of wild AMR resources, artificial cultivation is becoming more and more common. Reasonable planting methods of AMR could effectively improve land utilization efficiency and promote agricultural ecological balance. AMR produced in Zhejiang Province has been considered the best quality for hundreds of years because of its unique cultivation conditions and processing methods, called “Dao di” medicine. The quality of Anhui and Henan is the second, and Hebei is the worst [8]. According to reports, the lactone content, polysaccharide content, and essential oil content of AMR produced in Zhejiang Province are generally at a medium-to-high level [9,10]. There are four potential reasons for the differences in AMR quality from different regions. First, the difference of planting environment. For example, Zhejiang AMR is planted on the top of the mountain, while Anhui, Henan, and Hebei are all planted in plain areas. The large temperature difference between day and night on mountaintops has a significant impact on the accumulation of secondary metabolites in plant roots and stems. Second, the difference of cultivation methods, such as direct seeding of seeds, transplanting after seedling raising, and top pruning. Third, the difference of planting years. Zhejiang, Anhui, and Henan plant for two years, and Hebei plant for one year. Fourth, the processing methods of producing areas are different. Zhejiang adopts the traditional smoking method for drying, while other producing areas adopt machine drying or air drying [11].

In view of the enormous potential commercial value of AMR, some unscrupulous traders deliberately label inferior products as AMR and falsely advertise its geographical origin in order to obtain more profit. This deliberate fraud poses multiple challenges to the market supervision and quality control of AMR. Effective origin traceability of AMR is of great significance to ensure the quality and safety of herbal medicines and protect the health of consumers. From the perspective of public health, AMR origin traceability could help reduce medical accidents and disputes caused by quality problems of herbal medicines, and enhance the public’s trust in herbal medicines. Accurately identifying authentic AMR products can protect the rights and interests of consumers, promote the sustainable development of the AMR industry, maintain a fair competition order in the market, and promote the healthy development of the herbal medicine market. Moreover, it will also help to improve the overall quality and price of herbal medicines, bringing greater economic benefits to growers and enterprises. At present, Thin-Layer Chromatography (TLC) identification and extractives of 60% ethanol are stipulated in the quality standard of AMR in Chinese Pharmacopoeia (2020 edition), without assay, which could not control the quality of the product to a certain extent. Many scholars are working on quality control of AMR. Zhan et al. [12] quantitatively analyzed four components (AE I, AE II, AE III, and AO) in 18 batches of AMR samples with quantitative analysis of multi-components by single markers and established HPLC fingerprints. The results showed that these four components were significantly different from each other and there was no correlation between the contents of the four components in AMR. Therefore, only determining the content of one component is insufficient for quantitative analysis of AMR. It is suggested that determining the sum contents of four components and combining high-performance liquid chromatography (HPLC) fingerprints to control the quality of AMR. Furthermore, there are several instrumental analysis methods for AMR authenticity and origin traceability such as near-infrared spectroscopy [13], hyperspectral imaging [14], mineral element analysis based on inductively coupled plasma mass spectrometry combined with chemometrics [15], dual-channel colorimetric sensors based on metal ions [16], and so on. All these methods require professional operation and complicated analysis process. Therefore, it is necessary to develop a rapid, accurate, and precise method to control the quality of AMR more comprehensively.

Sensing techniques are a representative application of artificial intelligence applied to the quality evaluation of herb medicine, with the advantage of collecting integral and comprehensive information based on chemical composition [17]. The color, smell, and taste of the external characteristics of products are inevitably related to the internal chemical components. Electronic noses (E-nose) and colorimeters are the representative technologies of sensing techniques, in which E-noses are multi-sensory devices that employ more than one sensitive gas sensor to recognize complex mixtures of volatile organic compounds using pattern recognition techniques and artificial intelligence algorithms [18]. They are based on the advantages of simplicity and speed combined with machine learning algorithms and have been widely used for quality evaluation and origin tracing of herbal medicines [19,20], flavor characterization of food products [21], disease diagnosis [22], and so on. Du [23] explored the impact of solid-state fermentation on the polysaccharide content and color parameters of Glycyrrhiza stems and leaves through computer vision chemical analysis. Their study revealed a strong correlation between the color and polysaccharide content, and the PCA analysis revealed that fermentation stages can be effectively distinguished based on color variables. Chen [24] utilized computer vision and ultra-fast gas phase electronic nose technology to examine the morphological, chromatic, and gustatory characteristics of Pericarpium Citri Reticulatae (PCR) samples derived from diverse sources. The integration of multivariate statistical analysis with the BP neural network algorithm was employed, resulting in a discrimination rate of 100% and enabling rapid differentiation of PCR samples from diverse sources. Jing [25] explored a completely simple and practicable approach to identify the origins of Magnoliae Officinalis Cortex on the basis of E-nose, electronic tongue, and chemical analysis. The above study shows that the E-nose has a powerful sensor array to capture the subtle odor of the product, and the colorimeter can obtain the objective color parameters of the sample, which, combined with machine learning algorithms, could quickly analyze the product quality. However, few studies have reported on the application of sensing techniques for origin traceability and quality evaluation of AMR.

This study aims to trace the origins of AMR based on sensing techniques. The objectives include the following: (1) Determine the eight chemical components of AMR from four origins, including extracts, essential oil, polysaccharides, AEs and AO, to clarify the differences in composition among different samples. (2) Obtain objective color parameters and odor information of AMR powder by colorimeter and E-nose. (3) Principal component analysis (PCA) extracts variables and visualizes data. (4) Three machine learning methods based on different algorithm types, Partial least-squares discriminant analysis (PLS-DA), back-propagation neural network (BPNN), and a support vector machine based on a particle swarm optimization algorithm (PSO-SVM), are used to establish the origin classification model.

## 2. Results and Discussion

### 2.1. Results of 60% Ethanol Extracts

**Figure 1:**
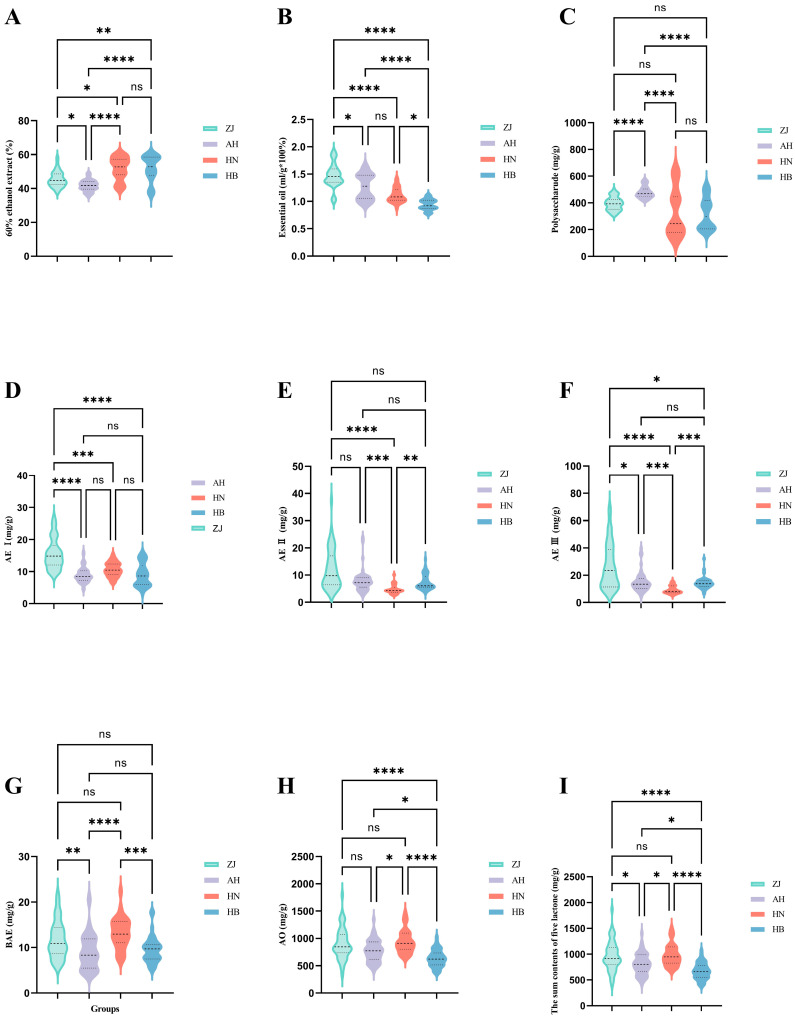
The violin plot of the content of physicochemical indices from different origins ((**A**) 60% ethanol extracts; (**B**) essential oil; (**C**) polysaccharide; (**D**) AE I; (**E**) AE II; (**F**) AE III; (**G**) BAE; (**H**) AO; (**I**) sum of AEs and AO) (* represents *p* < 0.05; ** represents *p* < 0.01; *** represents *p* < 0.001; **** represents *p* < 0.0001; ns represents *p* > 0.05).

In order to intuitively and comprehensively display the differences in the content of physicochemical indices from different origins, a violin plot is introduced for display, and the results are shown in Figure 1. The violin plot can display the concentration and dispersion of data, as well as the density distribution of the data. The wider its outline, the denser the distribution of data in this area; the narrower the outline, the sparser the distribution of the data in this area. The variable distribution diagram is shown in the violin plot whose middle line represents the median. The lower and upper lines of the plots represent the 25% and 75% quartiles of the tested data.

Figure 1A shows the results of the extract, from which it can be seen that there are significant differences in the extract content among the different origins, except for Hebei and Henan, where there is no difference. In terms of outline, the distributions of extract data in Hebei and Henan are relatively scattered, ranging from 36.16% to 60.43% and 40.01% to 60.57% (Table S3), respectively. The distributions of Zhejiang and Anhui are concentrated around the medians of 44.67% and 41.79%. In terms of mean value, the order of extract content from high to low is Hebei (51.77%) > Henan (51.70%) > Zhejiang (45.45%) > Anhui (41.68%). Extracts play an indispensable role in the quality evaluation of herb medicine, especially for situations where the content of other characteristic components is low and makes it difficult to represent the quality of herbs. To a certain extent, the extract content reflects the active ingredient content and efficacy of herbal medicine. However, it cannot fully represent all the medicinal ingredients and overall quality of herbs, and other characteristic components such as active ingredients need to be considered when evaluating the quality of herbal medicines. Therefore, the content of essential oil, polysaccharides, and lactones in AMR were selected to further evaluate its quality.

### 2.2. Results of Essential Oil

The essential oil is the main effective part of AMR, with a content of about 1.4% to 2.5% in its rhizome, which is closely related to its origin [26]. Modern pharmacological studies have shown that the essential oil of AMR has pharmacological effects such as regulating gastrointestinal motility [27], anti-inflammatory [28], anti-tumor, antioxidation, and antibacterial [29,30].

Essential oil extraction methods mainly include steam distillation, solvent extraction, pressing, ultrasonic extraction, supercritical fluid extraction, etc. Among them, steam distillation is the most commonly used method, and the obtained essential oil content is usually high. The solvent extraction method is suitable for some herbs that are not easily extracted by steam distillation. Due to the use of organic solvents, other fat-soluble components in the herbs will also be extracted at the same time, resulting in more impurities in the obtained essential oil. The pressing method is suitable for some herbs containing more volatile oil, such as fresh orange and lemon peel. Because of the high impurity content of the obtained essential oil, its application is limited. Therefore, in this paper, we chose the steam distillation method to extract essential oil of AMR, which is easy to operate and environmentally friendly [31,32]. The results are shown in Figure 1B and Table S3. There are significant differences in essential oil contents among the different origins, except for Anhui and Henan Provinces, where there is no difference. In terms of outline, the distributions of essential oil data in Zhejiang and Anhui are relatively scattered, ranging from 1.020% to 1.920% and 0.920% to 1.680, respectively. The distributions of Hebei and Henan are concentrated around the medians of 0.920% and 1.080%. In terms of mean value, the order of essential oil content from high to low is Zhejiang (1.472%) > Anhui (1.268%) > Henan (1.111%) > Hebei (0.9423%). This is consistent with previous research findings [33].

### 2.3. Results of Polysaccharide Content

The calibration curve result shows a good linearity of glucose content within 0.0106~0.1065 mg/mL, the regression equation is y = 4.8715x + 0.0752, and the coefficient of determination (R^2^) is 0.9997. The RSD of the precision test is 0.307%, indicating that the precision of the instrument is good. The stability test showed an RSD of 1.194% for the absorbance over 240 min, indicating that the sample solution is stable over 240 min. The RSD of the repeatability test is 2.525%, indicating that the method is well repeatable. The average recovery rate of the sample was 103.77%, with an RSD of 1.507%, indicating that the method is accurate.

The results of AMR polysaccharide content are shown in Figure 1C and Table S3. The polysaccharide content in Anhui is significantly different from the other three origins, while there is no significant difference among the other three origins. In terms of outline, the distributions of polysaccharide data in Henan and Hebei are relatively scattered, ranging from 133.0 mg/g to 646.0 mg/g and 177.4 mg/g to 530.4 mg/g, respectively. The distributions of Zhejiang and Anhui are concentrated around the medians of 393.3 mg/g and 470.0 mg/g. In terms of mean value, the order of polysaccharide content from high to low is Anhui (478.9 mg/g) > Zhejiang (394.4 mg/g) > Henan (334.1 mg/g) > Hebei (319.7 mg/g).

Polysaccharides widely exist in herbal medicines, and modern pharmacology has shown that polysaccharides are the important material basis for herbal medicines to exert their unique therapeutic effects, as well as the main active ingredients of plant antioxidants. Zhang [10] determined the content of AMR polysaccharide from Zhejiang, Hunan, and Anhui Provinces and studied differences in anti-fatigue properties of AMR polysaccharides from these three regions. The results showed that AMR produced in Zhejiang had the highest polysaccharide content and the best anti-fatigue effect. ZHU [34] analyzed the polysaccharide content and antioxidant activity of AMR from Zhejiang, Anhui, Hebei, and other producing areas, and found that the polysaccharide content of AMR from Zhejiang was the highest, while that from Hebei was the lowest. The antioxidant activity test showed that there were significant regional differences in the antioxidant activity of AMR, but the antioxidant activity was not related to the content of polysaccharides, and may be closely related to the composition and structure of polysaccharides. Most of the available reports on quality control studies of AMR have focused on AO and lactone constituents. As an important constituent of AMR, polysaccharides are the main material basis for its anti-fatigue effect, and it has pharmacological effects such as immunomodulatory, anti-tumor, gastroprotection and intestinal health promotion, hepatoprotective effect, and hypoglycemic activity [35]. Therefore, it is necessary to take it as a factor in evaluating the quality of AMR.

### 2.4. Results of HPLC

**Figure 2:**
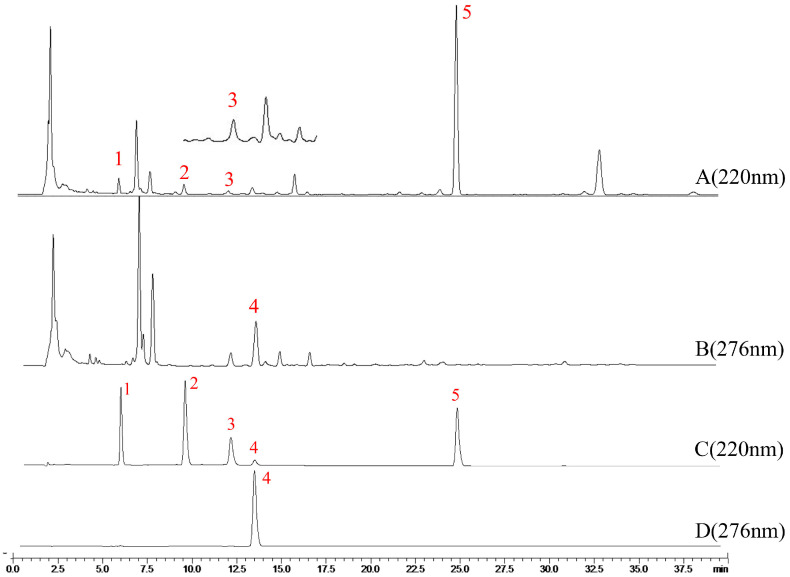
The typical chromatograms of AMR sample (A,B) and five standards (C,D). The peaks of 1, 2, 3, 4, and 5 represent AE III, AE II, BAE, AE I, and AO, respectively.

A robust HPLC method was developed and used to analyze all the sample solutions. As shown in Figure 2, the retention times of the four AEs and AO in the AMR sample solution were consistent with those of the reference solution. Furthermore, these five components were baseline separated and could be accurately determined by an external standard method. The validity of the HPLC method was carefully evaluated before the sample testing. The regression equation, the coefficient of determination (R^2^), and the linear range of five standard substances were given in Table 1 and indicated that the HPLC method was validated and applicable for sample analysis.

The results of five components’ contents are shown in Figure 1 and Table S3. The AE I content in Zhejiang is significantly different from the other three origins, while there is no significant difference among the other three origins. In terms of outline, the distributions of AE I data in Zhejiang and Hebei are relatively scattered, ranging from 9.010 mg/g to 26.85 mg/g and 3.240 mg/g to 15.87 mg/g, respectively. The distributions of Anhui and Henan are concentrated around the medians of 8.470 mg/g and 10.46 mg/g. In terms of mean value, the order of AE I content from high to low is Zhejiang (15.59 mg/g) > Henan (10.67 mg/g) > Hebei (9.050 mg/g) > Anhui (9.031 mg/g).

The AE II content in Henan is significantly different from the other three origins, while there is no significant difference among the other three origins. In terms of outline, the distributions of AE II data in Zhejiang and Anhui are relatively scattered, ranging from 3.080 mg/g to 37.34 mg/g and 2.500 mg/g to 23.85 mg/g, respectively. The distributions of Henan and Hebei are concentrated around the medians of 4.310 mg/g and 6.070 mg/g. In terms of mean value, the order of AE II content from high to low is Zhejiang (12.18 mg/g) > Anhui (7.899 mg/g) > Hebei (7.505 mg/g) > Henan (4.786 mg/g).

There are significant differences in the AE III content among the different origins, except for Anhui and Hebei, where there is no difference. In terms of outline, the distributions of AE III data in Zhejiang and Anhui are relatively scattered, ranging from 7.110 mg/g to 67.23 mg/g and 4.670 mg/g to 39.70 mg/g, respectively. The distributions of Henan and Hebei are concentrated around the medians of 8.260 mg/g and 13.80 mg/g. In terms of mean value, the order of AE III content from high to low is Zhejiang (26.54 mg/g) > Anhui (15.29 mg/g) > Hebei (14.45 mg/g) > Henan (9.372 mg/g).

There are significant differences in the content of BAE between Zhejiang and Anhui, Anhui and Henan, and Henan and Hebei. In terms of profile, the content of BAE is relatively scattered in all four origins. In terms of mean value, the order of BAE content from high to low is Henan (13.26 mg/g) > Zhejiang (11.72 mg/g) > Hebei (9.552 mg/g) > Anhui (9.020 mg/g).

There are significant differences in AO content between Hebei and the other three producing areas, as well as between Anhui and Henan, while there is no significant difference between Zhejiang and Anhui, or Zhejiang and Henan. In terms of profile, the content of AO is relatively scattered in all four origins. In terms of mean value, the order of AO content from high to low is Henan (940.3 mg/g) > Zhejiang (905.0 mg/g) > Anhui (770.2 mg/g) > Hebei (633.6 mg/g).

For the sum of the five components, there are significant differences among the different origins except for Zhejiang and Henan, where there is no difference. In terms of profile, the sum of the five components is relatively scattered in all four origins. In terms of mean value, the order of the sum of the five components from high to low is Henan (978.4 mg/g) > Zhejiang (971.0 mg/g) > Anhui (811.4 mg/g) > Hebei (674.2 mg/g).

Studies have reported that AO and AEs are unstable, among which AO is extremely unstable and can transform into AEs [12,36], which may be one of the reasons why the quality of AMR is difficult to control. The results of the above content determination revealed that any single component could not be distinguished from the origins. The next step will be to combine chemometrics for further analysis.

### 2.5. PCA Analysis of Physicochemical Content

PCA is an unsupervised method for compressing data space and orthogonalizing the original data matrix into a few PC scores to visually represent the characteristics of the multivariate data in a lowdimensional space, which can obtain the chemical variation profile of all samples.

**Figure 3:**
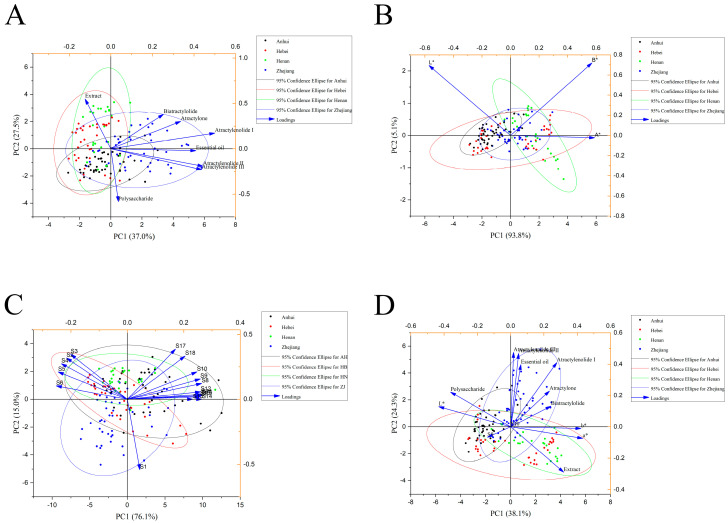
The biplot of PCA ((**A**) physicochemical indices; (**B**) color parameters; (**C**) maximum response value of E-nose; (**D**) fusion data of physicochemical, color parameters, and E-nose).

Figure 3A demonstrates the results of PCA of physicochemical indexes, and the variance contributions of PC1 and PC2 are 37.0% and 27.5%, respectively, explaining 64.5% of the total variance. It can be seen that the Zhejiang samples are almost in the positive semiaxis of PC1, which is positively correlated with essential oil, AEs, and AO. The Anhui, Henan, and Hebei samples are cross-distributed in the negative semiaxis of PC1, while the extract is positively correlated with Hebei and Henan, and the polysaccharide is positively correlated with Anhui.

In order to further compare the quality of AMR, the PCA total factor scores (F values) of 152 batches of AMR from different origins were obtained and ranked, and the larger the F-value, the better the quality. Among the samples with F-values ranking in the top 50, there are 23 batches from Zhejiang (8 batches from Anhui, 15 batches from Henan, and 4 batches from Hebei), accounting for 46% of the total number of samples in the top 50 (The F-values are shown in Table S4). The above data indicate that, in terms of chemical composition, the quality of Zhejiang AMR is generally better.

### 2.6. Color Measurement Analysis

**Figure 4:**
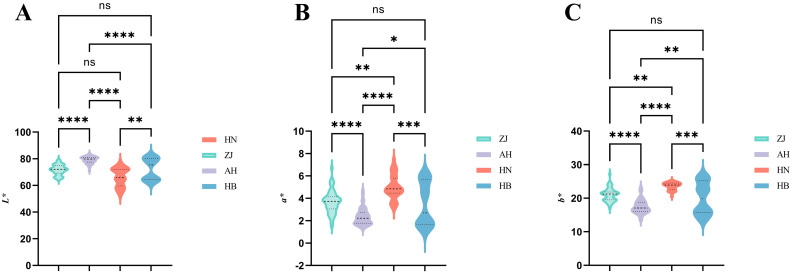
The violin plot of the color parameters from different origins ((**A**) L*; (**B**) a*; (**C**) b*) (* represents *p* < 0.05; ** represents *p* < 0.01; *** represents *p* < 0.001; **** represents *p* < 0.0001; ns represents *p* > 0.05).

The color parameters (L*, a*, and b*) of AMR were obtained by colorimeter. In order to quantify the color differences between different origins of AMR, we calculated the color parameter reference ranges and performed the Kruskal–Wallis test with significant difference by Duncan’s multiple tests. The results are shown in Figure 4 and Table S3.

In the CIE L*, a*, b* color coordinates, L* represents brightness, a* represents red and green, and b* represents yellow and blue, respectively. When a* is greater than 0, the color is red tone, and when b* is greater than 0, the color is yellow tone [37]. As can be seen from Figure 4 and Table S5, except for Zhejiang and Henan and Zhejiang and Hebei, where there is no significant difference in L*, the rest of the origins have a significant difference between each other, among which Anhui has the largest L*, indicating that its powder is the brightest. On the red–green coordinate, there is no significant difference between Zhejiang and Hebei, while there are significant differences between the rest of the origins. Among them, Henan has the largest a*, indicating that AMR powder of Henan has the deepest red tone. Likewise, on the yellow–blue coordinate, there is no significant difference between Zhejiang and Hebei, while there are significant differences between the rest of the origins. Among them, Henan has the largest b*, indicating that AMR powder of Henan has the deepest yellow tone.

The PCA result is shown in Figure 3B. It is disappointing that neither the Kruskal–Wallis test nor PCA could distinguish different origins.

### 2.7. Odor Information Analysis

The maximum response values of 18 sensors of the E-nose were selected for PCA analysis, and the result is shown in Figure 3C. The variance contributions of PC1 and PC2 are 76.1% and 15.0%, respectively, explaining 91.1% of the total variance. It can be seen that the Zhejiang samples are almost completely in the negative semiaxis of PC2, which is positively correlated with S1, representing oxidizing gas. Most of the samples from Anhui, Henan, and Hebei are distributed in the positive semiaxis of PC2. Among them, most of the samples from Hebei are related to sensors S2, S3, S4, S5, and S6, representing ammonia, organic amines, ethanol, hydrogen sulfide, propane, and butane, respectively. Most of the samples from Anhui are related to the rest of the S7 to S18 sensors, representing organic solvent, hydrocarbons, methane, fluorine, aromatic compounds, polar compound, alcohol, aldehyde compounds, and chlorinated compounds, respectively.

In view of the poor PCA results, we attempted to fuse physicochemical, color parameters, and E-nose data to perform PCA analysis, the result is shown in Figure 3D. The variance contributions of PC1 and PC2 are 38.1% and 24.3%, respectively, explaining 62.4% of the total variance. Obviously, the fusion data did not obtain more definitive results.

PCA is an unsupervised classification method that cannot effectively distinguish the origin of AMR. In the next step, the supervised classification method will be used to further analyze.

### 2.8. Qualitative Identification of Geographical Origins of AMR Based on Machine Learning

**Figure 5:**
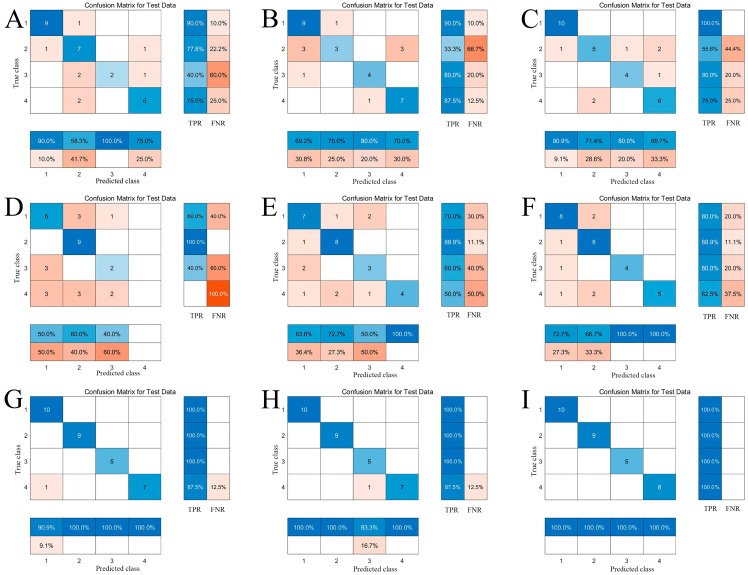
The confusion matrices of models ((**A**) (PLS-DA), (**B**) (BPNN), (**C**) (PSO-SVM): physicochemical content models; (**D**) (PLS-DA), (**E**) (BPNN), (**F**) (PSO-SVM): color parameter models; (**G**) (PLS-DA), (**H**) (BPNN), (**I**) (PSO-SVM): E-nose models). (1, 2, 3 and 4 represent Zhejiang, Anhui, Henan, and Hebei, respectively.) (The blue color represents true positive rate (TPR), the darker the blue color, the higher the TPR. Orange represents false negative rate (FNR), the darker the orange color, the higher the FNR).

The content of eight physicochemical indices, color parameters, and E-nose response values were taken as variables, and PLS-DA, BPNN, and PSO-SVM models were carried out with MATLAB 2022b software to distinguish the geographical origins of AMR (Figure 5). PLS-DA is a linear regression analysis method that can effectively solve the problem of covariance between the variables at high-latitude wavelengths. It has unique advantages in dealing with small samples and multivariate regression problems, and it has been widely used in the field of classification and regression [38]. BPNN is a multilayer feed-forward neural network, which is trained by an error back-propagation algorithm, capable of dealing with nonlinear problems, and widely used in the field of pattern recognition [39]. The PSO algorithm is a classical global optimization algorithm that finds the optimal solution by simulating the behavior of organisms such as birds or fishes [40]. SVM is a common machine learning algorithm that performs classification by identifying the boundary with the largest interval between various data points [41]. The PSO-SVM combines the global search capability of PSO with the classification performance of SVM, and is capable of finding the best classification hyperplane in a high-dimensional dataset. As classical classification algorithms, PLS-DA, BPNN, and SVM have been widely used in classification and regression prediction in various fields [42,43,44].

In the three models, 1, 2, 3, and 4 represent ZJ, AH, HN, and HB, respectively. All samples were divided into training and validation sets in a ratio of 4:1, and the results were visualized by confusion matrices, which provides a clear picture of the number and proportion of each category that is correctly or incorrectly categorized. The accuracy, true positive rate (TPR), and false negative rate (FNR) were used to evaluate the predictive ability. In general, the excellent predictive ability is manifested by the large value of accuracy and TPR and small value of FNR.

It can be seen from Figure 5 and Table 2 that AMR from different origins cannot be distinguished only by the physicochemical content and color parameters, with a low accuracy and TPR. Among them, Zhejiang has the highest TPR in the physicochemical content classification model, and Anhui has the highest TPR in the color parameter classification model, which is consistent with the results of the Kruskal–Wallis test. Surprisingly, the E-nose can effectively distinguish AMR from different origins. No matter which model, the accuracy is over 90%, and the SVM model combined with the PSO algorithm has an accuracy rate of 100%.

The above results found that the physicochemical content and color parameters could not distinguish AMR from different geographical origins. The reason may be the irrational selection of physicochemical indexes. Although polysaccharides, essential oil, AEs, and AO are the main active components of AMR, they are structurally unstable and unsuitable for use as indexes for the classification of origins. Liquid chromatography–mass spectrometry (LC-MS) and gas chromatography–mass spectrometry (GC-MS) should be used to analyze the whole components of AMR from different origins in future studies, and to further search the differential compounds to combine with existing methods to enhance discrimination accuracy. The color of AMR is greatly influenced by the processing method. For example, Zhejiang uses the traditional smoking method for drying, while Anhui, Henan, and Hebei use the mechanical drying method, and some products in Hebei use sun drying. The drying method, temperature, and time all have an impact on the color of AMR [11]. Therefore, it is difficult to distinguish AMR from different origins by using color parameters alone. AMR has a fragrant aroma, with Zhejiang products having a unique fragrance, while other origins have a slightly different fragrance that humans could not accurately identify. The E-nose is based on a sensitive sensor array that can capture subtle odors. It is an important tool for analyzing the volatile compounds of products, which is designed to imitate the biological olfactory system based on the use of metal oxide sensors that can be applied to analyze the total volatile profile (not single compounds) [45]. Although the E-nose does not employ highly specific receptors, it is feasible for generating distinctive patterns for diverse gases as their fingerprints through suitable machine learning techniques, thereby effectively facilitating the identification of AMR origins.

### 2.9. Correlation Analysis

**Figure 6:**
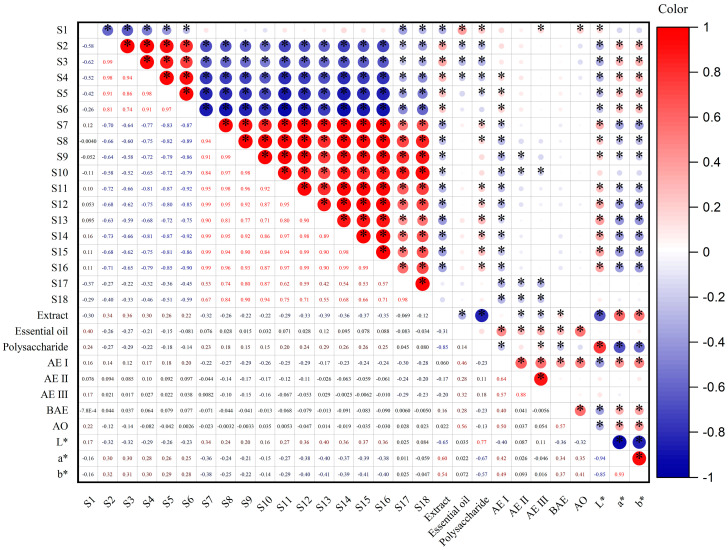
Spearman’s correlation analysis based on E-nose sensors, color parameters, and physicochemical content. (The color of a circle denotes the nature of the correlation, with 1 indicating a perfect positive correlation (dark red) and −1 indicating a perfect negative correlation (dark blue). Strong correlations and weak correlations are indicated by darker-colored circles and lighter-colored circles, respectively. * represents *p* < 0.05).

To further investigate the relationship between the E-nose sensors, color parameters, and physicochemical content, Pearson correlation analysis was conducted, and the results are presented in Figure 6. It can be seen that the extracts, polysaccharides, and AE I are significantly correlated with most of the sensors. For example, extracts have a significant negative correlation with sensors S1 and S7 to S16 and a significant positive correlation with sensors S2 to S6. Polysaccharides have a significant negative correlation with sensors S2, S3, S4, and S5 and a significant positive correlation with S1, S7, S8, and S11 to S16. AE I is significantly positively correlated with sensors S4, S5, and S6 and significantly negatively correlated with sensors S7 to S18. Meanwhile, essential oil and AEs are only correlated with a few sensors, such as essential oil having a significant positive correlation with sensor S1 and a significant negative correlation with sensors S2, S3, and S4, AE II having a significant negative correlation with sensors S9, S10, S17, and S18, AE III having a significant positive correlation with sensor S1 and a significant negative with sensors S10, S17, and S18, and AO only having a significant positive correlation with S1. Moreover, BAE has no correlation with any sensors.

The extracts, AE I, BAE, and AO have a significant negative correlation with L* and a significant positive correlation with a* and b*. Polysaccharides have a positive correlation with L* and a significant negative correlation with a* and b*.

In light of the correlation between E-nose sensors, color parameters, and physicochemical content, an attempt was made to develop a regression model for physicochemical content prediction using the aforementioned three algorithms (PLS-DA, BPNN, PSO-SVM) with E-nose sensors and color parameters as inputs and physicochemical content as outputs. However, the results were not satisfactory and the results are shown in Tables S6 and S7.

In addition, the content of essential oil has a significant positive correlation with AEs and AO, which is reasonable. AEs and AO are the main components in essential oil and increase with an increase in volatile oil content [26,28]. AE I has a significant positive correlation with AEs and AO, AE II has a significant positive correlation with AE III, and BAE has a significant positive correlation with AO. From the correlation analysis data, there is no mutual transformation between the five components of AEs and AO, and there may be other transformations, which need further study. This study indicates that these eight physicochemical indicators, as the main components that exert pharmacological effects, cannot be used as quality markers to distinguish different geographical origins, but they could be used as quality control indicators.

## 3. Materials and Methods

### 3.1. Chemical Reagents and Materials

Chemical reference standards of D-Glucose Anhydrous, AE I, AE II, and AE III were obtained from the National Institutes for Food and Drug Control (Beijing, China). Chemical reference standards of AO and Biatractylolide (BAE) were obtained from Shanghai yuanye Bio-Technology Co., Ltd. (Shanghai, China). HPLC-grade acetonitrile (99.95%) was purchased from Thermo Fisher Scientific Co., Ltd. (Shanghai, China). Analytical pure phenol (99.98%) and ethanol absolute (99.80%) were purchased from Fuchen (Tianjin, China) Chemical Reagent Co., Ltd. (Tianjin, China). Analytical pure sulfuric acid (98.08%) was from Beijing Tongguang Chemical Co. (Beijing, China). Purified water was purchased from Hangzhou Wahaha Group Co., Ltd. (Hangzhou, China). All 152 batches of AMR samples (numbered ZJ 1 to ZJ 46, AH 1 to AH 44, HN 1 to HN 27, HB 1 to HB 35) were collected from Zhejiang, Anhui, Henan, and Hebei Provinces in China, and the source information is listed in Table S1. The authentication of the samples was identified by Professor Yong-Hong Yan from the Department of Chinese Materia Medica of Beijing University of Chinese Medicine according to the morphological features.

### 3.2. Determination of 60% Ethanol Extracts

According to the Chinese Pharmacopoeia (2020 edition) hot-dip method of alcohol-soluble extracts (General Rule 2201), with 60% ethanol as the solvent, an amount of approximately 2.0 g AMR powder (AMR samples were ground to powder and sifted through a 50-mesh sieve) was accurately weighed in a 100 mL conical flask, had 100 mL of 60% (*v*/*v*) ethanol added, was sealed tightly, weighed, and left for 1 h. Then, the reflux condenser was connected to heat it, and kept it slightly boiling for 1 h. Then, the conical flask was sealed tightly and weighed again after cooling, making up for the lost weight with 60% (*v*/*v*) ethanol, filtered through a drying filter. The filtrate was accurately transferred 25 mL into an evaporating dish that was dried to constant weight, evaporated in a water bath, dried at 105 °C for 3 h, and quickly weighed accurately after cooling for 30 min in a dryer. Each sample was measured in triplicate and the average was taken.

### 3.3. Determination of Essential Oil

According to the Chinese Pharmacopoeia (2020 edition) General Principles 2204, “Determination of Essential Oil”, Method A, an amount of approximately 100.0 g AMR powder was accurately weighed in a 1000 mL round-bottomed flask. Then, we added 500 mL of water and connected the essential oil analyzer to the reflux condenser. Water was added to fill the scale of the essential oil analyzer from the top of the condenser tube, which was overflowed into the flask. We placed it in an electric heating jacket and slowly heated until it boiled, keeping it slightly boiling for 5 h (we investigated the extraction yield of essential oil at different heating times (3, 5, 7 h) and found that the extraction yield was highest when extracted for 5 h). Heating was stopped to leave it for a moment, then we opened the piston at the lower end of the measuring instrument, and slowly released the water until the upper end of the oil layer reached 5 mm above the scale 0 line. After leaving it for more than 1 h, we opened the piston to make the oil layer drop to its upper end exactly level with the 0 line of the scale. Lastly, we read the amount of essential oil, and calculated the content of essential oil in the sample (%).

### 3.4. Determination of Polysaccharide Content

#### 3.4.1. Preparation of Standard Solutions

For 5% phenol solution: An amount of approximately 5.0 g phenol was accurately weighed in a 100 mL brown volumetric flask, with water added to dissolve and make up the volume to the scale.

D-Glucose standard solution: An appropriate amount of D-Glucose Anhydrous was dissolved in water to prepare a glucose standard solution with a concentration of 0.1065 mg/mL.

Sample solution: An amount of approximately 0.50 g AMR powder was accurately weighed in a 250 mL round-bottomed flask and 100 mL of 80% (*v*/*v*) ethanol was added. The solution was treated with heat and reflux at 85 °C for 1.5 h, and filtered while hot. Filter residue with filter paper was placed in the original flask, 150 mL of water added, and the mixture was heated at reflux at 90 °C for 3 h. The solution was filtered while hot, and a small amount of hot water should be used to wash the filter, after which the filtrate and washing solution should be combined. Then, we transferred the solution into a 250 mL volumetric flask after it cooled, and diluted to the mark with water. Lastly, the solution obtained in the previous step was accurately transferred in a measure of 1 mL to a 25 mL volumetric flask and diluted with water to the mark to obtain the sample solution.

#### 3.4.2. Calibration Curve

Amounts of 20, 50, 80, 110, 140, 170, and 200 μL of D-Glucose standard solution were accurately transferred into a 2 mL centrifuge tube, respectively. Subsequently, 200 μL of water, 200 μL of a 5% phenol solution and 700 μL of sulfuric acid should be added to each tube. Then, the tubes should be shaken well and quickly, and kept warm in an 80 °C water bath for 10 min. Following this, the tubes should be removed and cooled in a cold-water bath for 5 min to stop the reaction. Finally, 150 μL of each reaction solution should be transferred into a 96-well microtiter plate accurately, and the absorbance measured at a wavelength of 490 nm. The calibration curve could be calculated with absorbance as the *y*-axis and glucose concentration as the *x*-axis.

#### 3.4.3. Sample Determination

An amount of 200 μL of sample solution was accurately transferred into a 2 mL centrifuge tube, and followed the method “3.4.2 Calibration curve” to determine the absorbance starting from “200 μL of water, 200 μL of a 5% phenol solution”. The concentration of glucose in the sample solution (mg/mL) was calculated based on the calibration curve. Each sample was measured in triplicate and the average was taken.

#### 3.4.4. Validation of the Polysaccharide Quantification Method

According to the guidelines for analytical method validation in Chinese Pharmacopoeia (2020 edition), the precision of the BioTek instrument (Highland Park, Winooski, VT, USA) was evaluated by standard solution six times with successive injections. The stability test of sample solutions was performed by analyzing the same sample solution at 0, 30, 60, 120, 180, and 240 min. The repeatability was determined by preparing six sample solutions independently and calculating the RSDs (Relative Standard Deviation, RSD) of the contents. The recovery test was conducted to evaluate the accuracy of this method, which was performed by adding a known amount of D-Glucose standard solution to the six AMR solutions and calculating the recovery rate and the RSDs.

### 3.5. HPLC Determination

#### 3.5.1. Preparation of Standard Solutions

Stock standard solutions of AE I, AE II, AE III, BAE, and AO were prepared by dissolving appropriate amounts of reference substances into methanol. An appropriate volume of each stock solution was taken to prepare mixed standard solution (MSS), in which the concentration of each component was 0.522, 0.528, 0.574, 0.882, and 1.322 mg/mL, respectively. Then, MSS was diluted with methanol to obtain a series of solutions with different concentrations in order to establish the calibration curve. All the solutions were filtered by 0.45 μm microporous filtering film before analysis and stored at 4 °C away from light.

#### 3.5.2. Preparation of Sample Solutions

AMR powders of 1.0 g were extracted with 20 mL methanol in a conical flask with a stopper by an ultrasonic cleaner (250 W, 40 kHz) for 30 min at room temperature (the ultrasonic cleaner’s frequency and power were verified as consistent during experiments.). In order to make up for the weight loss during ultrasonic extraction, additional methanol was added to the extract solution. Then, the AMR sample solution was filtered by 0.42 μm microporous filtering film before analysis and stored at 4 °C away from light.

#### 3.5.3. HPLC Conditions

The HPLC analysis was performed on a DGU-403 LC series–diode array detector (DAD) system. A Kromasil 100-5C18 (4.6 mm × 250 mm, 5 μm, Bohus, Sweden) was used to separate components, using water (A)—acetonitrile (B) as the mobile phase, and the flow rate was kept at 1.0 mL/min. The gradient elution program was as follows: 0–10 min, 68% B, 10–15 min, 68–80% B, 15–20 min, 80–85% B, 20–25 min, 85–90% B, 25–40 min, 90% B. The detection wavelength was set at 220 nm for AE II, AE III, BAE, and AO, and 276 nm for AE I. The column temperature was maintained at 30 °C. The injection volume was 10 μL.

#### 3.5.4. Validation of the HPLC Quantification Method

According to the guidelines for analytical method validation in Chinese Pharmacopoeia (2020 edition), the linearity regression curves for each component were obtained by plotting the peak areas against the concentrations of each component standard solution. The precision of the HPLC instrument was evaluated by MSS six times with successive injections. The stability test of sample solutions was performed by analyzing the same sample solution at 0, 2, 4, 8, 12, 24, and 48 h. The repeatability was determined by preparing six sample solutions independently and calculating the RSDs of the contents. The recovery test was conducted to evaluate the accuracy of this method. The recovery test of a solution was performed by adding a known amount of AE I, AE II, AE III, BAE, and AO standard solution, respectively, to the six AMR solutions and calculating the recovery rate and the RSDs.

### 3.6. Color Parameter Determination

The AMR powder color was determined by a CM-5 colorimeter (Konica Minolta Holdings, Inc. Chiyoda-ku, Tokyo, Japan). The colorimeter was calibrated with a black and white box before measurement. An appropriate amount of the powder was added to a 30 mm color measuring dish, starting and ending wavelength range: 350–750 nm, scanning speed: 600 nm/min, slit width: 1 nm, illumination source: D65, and field of view: 10°. The AMR colors are represented by L*, a*, and b*, where *L* represents brightness, *a* represents red and green, and *b* represents yellow and blue. Each sample was measured in triplicate and the average was taken.

### 3.7. E-Nose Analysis

An α-Fox4000 E-nose (Alpha MOS, Co., Ltd., Toulouse, France) with 18 metal-oxide gas sensors was employed to obtain the odor information of AMR. The names and response characteristics of each sensor are presented in Table S2. The E-nose was self-checked and the sensor array was preheated for 2–3 h before each sampling experiment. Processed pure air was used as carrier gas to clean the sensor array, making the signal response back to baseline. AMR powder (0.4 g) was sealed into a 20 mL vial and incubated for 10 min at 45 °C (250 rpm). The temperature and volume of injection were set at 45 °C and 1500 μL. The flow rate of carrier gas was 150 mL/min. The data acquisition interval and data acquisition cycle were 1 s and 120 s, respectively. Every AMR sample was continuously sampled 3 times, and the maximum response values of the E-nose sensor were extracted and used for further analysis.

### 3.8. Data Analysis

In order to clarify the differences in physicochemical indices and color parameter among different origins, the Kruskal–Wallis test with significant difference by Duncan’s multiple tests (*p* < 0.05) was implemented through GraphPad Prism software (version 9.4.0). The PCA of physicochemical contents, colorimetric parameters, E-nose response values, and the fusion information of the three of them were performed by Origin 2022 software, respectively, to achieve initial classification and data visualization. The Spearman’s correlation analysis of physicochemical contents, E-nose, and color parameters was achieved through Origin 2022 software to explore their correlation. Qualitative origin classification models and quantitative content prediction models based on PLS-DA, BPNN, and PSO-SVM were built by MATLAB 2022b software (MathWorks Inc., Natick, MA, USA), to further investigate the application of sensing techniques in the origin traceability and quality evaluation of AMR.

## 4. Conclusions

Based on the chemical content analysis of extracts, essential oils, polysaccharides, AEs, and AO, it has been demonstrated that the quality of AMR from Zhejiang was superior overall, but only relying on these eight chemical components could not distinguish AMR from different geographical origins. Despite significant differences in the color parameters of AMR powder from different origins, the classification model established by machine learning methods cannot distinguish the different origins of AMR. The E-nose employs a combination of three machine learning methods, PLS-DA, BPNN, and PSO-SVM, enabling the classification of AMR from different origins with an accuracy of 100%. In terms of poor quantitative content prediction models, in follow-up studies we can further extract features from all 120 s data of the E-nose (not only maximum response values) or fuse them with color parameters to explore highly correlated features with these contents to establish prediction models, which may achieve better results.

In the future, portable E-nose devices equipped with only one or a few sensors may be able to be widely used in the market for easy detection of the geographical origin of AMR products. In subsequent studies, screening and optimization of E-nose sensor arrays will be one of the research directions. Undoubtedly, our study provides a research foundation for rapid geographical origin traceability of AMR and manufacturing portable E-nose devices.

## Figures and Tables

**Table 1:** Methodological investigation parameters of the HPLC results (*n* = 6).

Reference Substances	Regression Equation	R^2^	Linear Range (μg/mL)	Precision (RSD%)	Stability (RSD%)	Repeatability (RSD%)	Recovery	
							Mean	(RSD%)
AE I	y = 53,325,579x + 41,087	0.9999	2.088~417.6	0.4990	2.3568	1.218	104.0	1.963
AE II	y = 48,260,147x + 44,726	0.9999	2.112~422.4	0.9870	2.705	1.729	100.3	2.242
AE III	y = 25,727,546x + 156,284	0.9995	2.296~459.2	0.4075	1.882	1.164	104.7	2.792
BAE	y = 24,369,038x + 21,748	1.0000	3.528~705.6	1.386	2.4847	2.261	102.7	2.290
AO	y = 15,782,192x − 27,003	1.0000	5.288~1057.6	0.1862	0.2239	2.685	105.9	2.532

**Table 2:** Comparison of accuracy from PLS-DA, BPNN, and PSO-SVM models (%).

Models	Physicochemical Indices		Color Parameters		E-Nose	
	Training Set	Test Set	Training Set	Test Set	Training Set	Test Set
PLS-DA	77.50	75.00	55.83	53.13	90.00	96.88
BPNN	89.17	71.88	79.17	68.75	98.33	96.88
PSO-SVM	100.0	78.13	78.33	78.13	100.0	100.0

## Data Availability

Data are contained within the article and Supplementary Materials.

## Supplementary Materials

The following supporting information can be downloaded at: https://www.mdpi.com/article/10.3390/molecules29214991/s1, Table S1: The AMR sample information; Table S2: Detailed information of 18 metal oxide sensors; Table S3: The physicochemical content descriptive statistics of the Kruskal–Wallis test; Table S4: Scores of principal component and comprehensive principal component values; Table S5: The color parameter descriptive statistics of the Kruskal–Wallis test; Table S6. The results of the physicochemical content prediction model based on electronic nose; Table S7: The results of physicochemical content prediction models based on color parameters.
