# Supplementary material for: Geographical Origin Traceability of Atractylodis Macrocephalae Rhizoma Based on Chemical Composition, Chromaticity, and Electronic Nose
Source: Molecules. 2024 Oct 22;29(21):4991. doi: 10.3390/molecules29214991 (PMC11547543; doi:10.3390/molecules29214991)
Supplement: Supplementary file 1 [file molecules-29-04991-s001.zip › molecules-3236705-supplementary.pdf]

Geographical Origin Traceability of *Atractylodis Macrocephalae* Rhizoma based on Chemical Composition, Chromaticity and Electronic-nose

Rui-Qi Yang, Yu-shi Wang, Jia-yu Wang, Xing-yu Guo, Yuan-yu Zhao, Ke-yao Zhu, Xin-tian Zhu, Hui-Qin Zou*, Yong-Hong Yan*

School of Chinese Materia Medica, Beijing University of Chinese Medicine, Beijing, China.

*Corresponding authors: zouhuiqin_bucm@sina.cn (Hui-Qin Zou),
lxdyyh@yeah.net (Yong-Hong Yan).

Table S1. The AMR samples information

Sample Number	Origin	Sample Number	Origin
ZJ-1	Taizhou, Zhejiang Province	ZJ-39	Jinhua, Zhejiang Province
ZJ-2	Taizhou, Zhejiang Province	ZJ-40	Jinhua, Zhejiang Province
ZJ-3	Taizhou, Zhejiang Province	ZJ-41	Jinhua, Zhejiang Province
ZJ-4	Taizhou, Zhejiang Province	ZJ-42	Jinhua, Zhejiang Province
ZJ-5	Taizhou, Zhejiang Province	ZJ-43	Shaoxing, Zhejiang Province
ZJ-6	Taizhou, Zhejiang Province	ZJ-44	Shaoxing , Zhejiang Province
ZJ-7	Taizhou, Zhejiang Province	ZJ-45	Shaoxing, Zhejiang Province
ZJ-8	Shaoxing, Zhejiang Province	ZJ-46	Shaoxing , Zhejiang Province
ZJ-9	Shaoxing , Zhejiang Province	AH-1	Bozhou, Anhui Province
ZJ-10	Shaoxing, Zhejiang Province	AH-2	Bozhou, Anhui Province
ZJ-11	Shaoxing , Zhejiang Province	AH-3	Bozhou, Anhui Province
ZJ-12	Shaoxing, Zhejiang Province	AH-4	Bozhou, Anhui Province
ZJ-13	Shaoxing , Zhejiang Province	AH-5	Bozhou, Anhui Province
ZJ-14	Shaoxing, Zhejiang Province	AH-6	Bozhou, Anhui Province
ZJ-15	Shaoxing , Zhejiang Province	AH-7	Bozhou, Anhui Province
ZJ-16	Shaoxing, Zhejiang Province	AH-8	Bozhou, Anhui Province
ZJ-17	Shaoxing , Zhejiang Province	AH-9	Bozhou, Anhui Province
ZJ-18	Shaoxing, Zhejiang Province	AH-10	Bozhou, Anhui Province
ZJ-19	Shaoxing , Zhejiang Province	AH-11	Bozhou, Anhui Province
ZJ-20	Shaoxing, Zhejiang Province	AH-12	Bozhou, Anhui Province
ZJ-21	Shaoxing , Zhejiang Province	AH-13	Bozhou, Anhui Province
ZJ-22	Shaoxing, Zhejiang Province	AH-14	Bozhou, Anhui Province
ZJ-23	Shaoxing , Zhejiang Province	AH-15	Bozhou, Anhui Province
ZJ-24	Shaoxing, Zhejiang Province	AH-16	Bozhou, Anhui Province
ZJ-25	Shaoxing , Zhejiang Province	AH-17	Bozhou, Anhui Province
ZJ-26	Jinhua, Zhejiang Province	AH-18	Bozhou, Anhui Province
ZJ-27	Jinhua, Zhejiang Province	AH-19	Bozhou, Anhui Province
ZJ-28	Jinhua, Zhejiang Province	AH-20	Bozhou, Anhui Province
ZJ-29	Jinhua, Zhejiang Province	AH-21	Bozhou, Anhui Province
ZJ-30	Jinhua, Zhejiang Province	AH-22	Bozhou, Anhui Province
ZJ-31	Jinhua, Zhejiang Province	AH-23	Bozhou, Anhui Province
ZJ-32	Jinhua, Zhejiang Province	AH-24	Bozhou, Anhui Province
ZJ-33	Jinhua, Zhejiang Province	AH-25	Bozhou, Anhui Province
ZJ-34	Jinhua, Zhejiang Province	AH-26	Bozhou, Anhui Province
ZJ-35	Jinhua, Zhejiang Province	AH-27	Bozhou, Anhui Province
ZJ-36	Taizhou, Zhejiang Province	AH-28	Bozhou, Anhui Province
ZJ-37	Taizhou, Zhejiang Province	AH-29	Bozhou, Anhui Province
ZJ-38	Taizhou, Zhejiang Province	AH-30	Bozhou, Anhui Province

Continued Table S1. The AMR samples information

Sample Number	Origin	Sample Number	Origin
AH-31	Bozhou, Anhui Province	HN-25	Shangqiu, Henan Province
AH-32	Bozhou, Anhui Province	HN-26	Shangqiu, Henan Province
AH-33	Bozhou, Anhui Province	HN-27	Shangqiu, Henan Province
AH-34	Bozhou, Anhui Province	HB-1	Dingzhou, Hebei Province
AH-35	Bozhou, Anhui Province	HB-2	Dingzhou, Hebei Province
AH-36	Bozhou, Anhui Province	HB-3	Dingzhou, Hebei Province
AH-37	Bozhou, Anhui Province	HB-4	Dingzhou, Hebei Province
AH-38	Bozhou, Anhui Province	HB-5	Dingzhou, Hebei Province
AH-39	Bozhou, Anhui Province	HB-6	Dingzhou, Hebei Province
AH-40	Bozhou, Anhui Province	HB-7	Anguo, Hebei Province
AH-41	Bozhou, Anhui Province	HB-8	Anguo, Hebei Province
AH-42	Bozhou, Anhui Province	HB-9	Anguo, Hebei Province
AH-43	Bozhou, Anhui Province	HB-10	Anguo, Hebei Province
AH-44	Bozhou, Anhui Province	HB-11	Anguo, Hebei Province
HN-1	Zhoukou, Henan Province	HB-12	Anguo, Hebei Province
HN-2	Zhoukou, Henan Province	HB-13	Anguo, Hebei Province
HN-3	Zhoukou, Henan Province	HB-14	Anguo, Hebei Province
HN-4	Zhoukou, Henan Province	HB-15	Anguo, Hebei Province
HN-5	Zhoukou, Henan Province	HB-16	Anguo, Hebei Province
HN-6	Zhoukou, Henan Province	HB-17	Anguo, Hebei Province
HN-7	Zhoukou, Henan Province	HB-18	Anguo, Hebei Province
HN-8	Zhoukou, Henan Province	HB-19	Anguo, Hebei Province
HN-9	Zhoukou, Henan Province	HB-20	Anguo, Hebei Province
HN-10	Zhoukou, Henan Province	HB-21	Anguo, Hebei Province
HN-11	Zhoukou, Henan Province	HB-22	Anguo, Hebei Province
HN-12	Zhoukou, Henan Province	HB-23	Anguo, Hebei Province
HN-13	Zhoukou, Henan Province	HB-24	Anguo, Hebei Province
HN-14	Zhoukou, Henan Province	HB-25	Anguo, Hebei Province
HN-15	Zhoukou, Henan Province	HB-26	Anguo, Hebei Province
HN-16	Shangqiu, Henan Province	HB-27	Anguo, Hebei Province
HN-17	Shangqiu, Henan Province	HB-28	Anguo, Hebei Province
HN-18	Shangqiu, Henan Province	HB-29	Anguo, Hebei Province
HN-19	Shangqiu, Henan Province	HB-30	Anguo, Hebei Province
HN-20	Shangqiu, Henan Province	HB-31	Anguo, Hebei Province
HN-21	Shangqiu, Henan Province	HB-32	Anguo, Hebei Province
HN-22	Shangqiu, Henan Province	HB-33	Anguo, Hebei Province
HN-23	Shangqiu, Henan Province	HB-34	Anguo, Hebei Province
HN-24	Shangqiu, Henan Province	HB-35	Anguo, Hebei Province

Table S2. Detailed information of 18 metal oxide sensors

Number	Type of sensors	Sensitive substance
S1	LY2/LG	Oxidizing gas
S2	LY2/G	Ammonia/organic amines, carbon monoxide
S3	LY2/AA	Ethanol
S4	LY2/GH	Ammonia/organic amines
S5	LY2/gCTL	Hydrogen sulfide
S6	LY2/gCT	Propane, butane
S7	T30/1	Organic solvent
S8	P10/1	Hydrocarbons, methane
S9	P10/2	Methane
S10	P40/1	Fluorine
S11	T70/2	Aromatic compounds
S12	PA/2	Ethanol, ammonia/organic amines
S13	P30/1	Polar compound (ethanol)
S14	P40/2	Heteroatoms/chlorides/aldehydes
S15	P30/2	Alcohol
S16	T40/2	Aldehyde compounds
S17	T40/1	Chlorinated compounds
S18	TA/2	Air

Table S3. The physicochemical content descriptive statistics of Kruskal-Wallis test

Project	Origins	ZJ	AH	HN	HB
60% ethanol extracts	Reference ranges (%)	37.86~58.47	35.17~49.13	40.01~60.57	36.16~60.43
	Mean (%)	45.45	41.68	51.70	51.77
	Mean ranks	71.25	43.20	104.6	103.6
	Kruskal-Wallis H	50.07			
	P value	< 0.0001			
Essential oil	Reference ranges (%)	1.02~1.92	0.92~1.68	0.88~1.42	0.78~1.12
	Mean (%)	1.472	1.268	1.111	0.9423
	Mean ranks	114.3	85.35	61.06	27.64
	Kruskal-Wallis H	82.16			
	P value	< 0.0001			
Polysaccharide	Reference ranges	311.8~499.5	414.5~575.5	133.0~646.0	177.4~530.4
	Mean (mg/g)	394.4	478.9	334.1	319.7
	Mean ranks	68.72	114.5	59.07	52.34
	Kruskal-Wallis H	49.07			
	P value	< 0.0001			
AE Ⅰ	Reference ranges	9.010~26.85	4.450~16.22	6.700~15.26	3.240~15.87
	Mean (mg/g)	15.59	9.031	10.67	9.050
	Mean ranks	116.4	52.91	75.91	54.16
	Kruskal-Wallis H	59.47			
	P value	< 0.0001			
AE Ⅱ	Reference ranges	3.080~37.34	2.500~23.85	2.630~10.09	3.800~15.89
	Mean (mg/g)	12.18	7.899	4.786	7.505
	Mean ranks	101.2	77.59	33.61	75.70
	Kruskal-Wallis H	40.19			
	P value	< 0.0001			
AE Ⅲ	Reference ranges	7.110~67.23	4.670~39.70	5.360~15.60	6.710~32.17
	Mean (mg/g)	26.54	15.29	9.372	14.45
	Mean ranks	103.2	74.95	32.78	77.06
	Kruskal-Wallis H	43.64			
	P value	< 0.0001			
BAE	Reference ranges	5.590~21.67	3.790~20.98	7.440~22.36	5.020~17.88
	Mean (mg/g)	11.72	9.020	13.26	9.552
	Mean ranks	86.75	57.42	106.6	63.80
	Kruskal-Wallis H	26.29			
	P value	< 0.0001			
AO	Reference ranges	335.5~1796	413.3~1346	667.3~1452	377.6~1018
	Mean (mg/g)	905.0	770.2	940.3	633.6
	Mean ranks	91.83	71.02	102.4	43.29
	Kruskal-Wallis H	35.50			
	P value	< 0.0001			

Table S4. Scores of principal component and comprehensive principal component value

Number	F1	F2	F	Rank	Number	F1	F2	F	Rank
ZJ-6	643.5	310.8	501.8	1	ZJ-37	364.8	51.39	231.2	39
HN-14	501.6	352.6	438.1	2	AH-5	362.2	53.91	230.8	40
ZJ-35	515.2	258.6	405.9	3	HN-6	269.3	176.0	229.5	41
HN-8	461.6	319.1	400.9	4	HN-18	343.9	74.04	228.9	42
HN-10	473.9	301.7	400.6	5	AH-19	346.2	69.06	228.1	43
ZJ-43	493.4	228.1	380.4	6	ZJ-28	317.8	104.2	226.8	44
ZJ-30	476.5	242.6	376.8	7	ZJ-32	340.2	64.50	222.7	45
ZJ-31	476.2	229.2	371.0	8	AH-15	355.2	41.99	221.7	46
ZJ-34	480.8	190.0	356.9	9	ZJ-18	311.8	95.91	219.8	47
AH-29	486.8	173.2	353.2	10	AH-16	350.8	37.85	217.5	48
HN-15	393.2	250.1	332.3	11	HB-25	277.5	136.1	217.3	49
HN-12	387.2	250.1	328.8	12	ZJ-19	320.3	75.99	216.2	50
HN-13	392.2	224.9	320.9	13	AH-37	359.2	21.90	215.5	51
ZJ-41	440.9	157.7	320.2	14	HB-21	267.4	141.8	213.9	52
HN-11	387.7	227.9	319.6	15	HN-21	330.5	54.16	212.7	53
ZJ-44	421.4	158.9	309.5	16	AH-23	357.2	6.332	207.7	54
HN-9	364.2	232.6	308.2	17	HB-20	261.9	133.8	207.3	55
ZJ-46	432.9	140.0	308.1	18	AH-25	352.9	8.230	206.0	56
HN-1	358.9	239.5	308.0	19	HN-7	241.4	157.7	205.7	57
HB-26	362.6	223.2	303.2	20	ZJ-25	328.9	37.80	204.9	58
ZJ-13	415.1	151.7	302.8	21	HN-19	317.3	53.25	204.8	59
AH-11	428.3	103.3	289.8	22	ZJ-26	291.9	80.10	201.6	60
ZJ-33	386.6	155.8	288.3	23	HB-24	261.2	121.0	201.5	61
ZJ-29	385.2	149.4	284.7	24	AH-35	323.8	32.28	199.6	62
ZJ-14	377.6	148.3	279.9	25	ZJ-38	314.7	44.33	199.5	63
HN-4	314.7	230.4	278.8	26	ZJ-27	287.6	80.39	199.3	64
HN-2	320.8	221.7	278.5	27	ZJ-12	345.3	1.488	198.8	65
ZJ-36	401.9	111.7	278.3	28	HB-23	250.5	128.1	198.4	66
HB-19	335.1	190.4	273.4	29	HN-20	308.7	44.71	196.2	67
AH-27	394.7	90.1	265.0	30	HB-4	310.7	29.84	191.0	68
AH-18	393.6	74.0	257.4	31	AH-24	347.8	-21.19	190.5	69
HN-3	284.2	206.0	250.8	32	HB-2	292.6	51.40	189.8	70
ZJ-42	373.4	80.8	248.7	33	AH-10	305.3	33.23	189.4	71
ZJ-5	367.7	85.2	247.3	34	ZJ-39	312.4	18.33	187.1	72
ZJ-24	372.9	75.4	246.2	35	HN-27	357.9	-43.36	186.9	73
HB-22	297.4	176.9	246.1	36	ZJ-11	328.2	-6.122	185.7	74
HN-5	278.	188.6	240.4	37	ZJ-40	300.1	31.08	185.5	75
ZJ-8	380.9	36.32	234.1	38	HB-33	237.6	111.9	184.0	76

Continued Table S4. Scores of principal component and comprehensive principal component value

Number	F1	F2	F	Rank	Number	F1	F2	F	Rank
HB-27	236.9	112.7	184.0	77	AH-43	280.4	-77.58	127.9	115
HB-1	298.6	28.09	183.3	78	HB-6	226.9	-8.809	126.5	116
AH-33	314.9	-4.509	178.8	79	HB-28	178.9	54.09	125.7	117
AH-30	307.3	4.777	178.4	80	HB-16	263.7	-60.92	125.4	118
AH-21	310.5	-0.7484	177.9	81	AH-14	248.0	-42.68	124.1	119
ZJ-10	320.3	-15.42	177.2	82	AH-1	240.4	-35.47	122.9	120
ZJ-23	305.6	-1.448	174.8	83	ZJ-1	241.7	-69.17	109.2	121
AH-4	286.3	17.41	171.7	84	AH-44	260.8	-99.15	107.4	122
HN-23	346.8	-65.79	171.0	85	AH-41	258.6	-96.73	107.2	123
ZJ-7	283.5	15.08	169.1	86	AH-8	256.4	-94.65	106.8	124
ZJ-20	293.4	0.3299	168.5	87	HB-17	244.3	-80.42	105.9	125
HB-29	224.6	88.63	166.6	88	HB-5	223.8	-53.94	105.5	126
HB-18	318.8	-39.67	166.0	89	HN-24	261.5	-108.1	104.0	127
AH-6	289.8	-0.8528	165.9	90	HB-14	200.9	-29.30	102.8	128
AH-28	304.5	-24.55	164.3	91	HN-26	273.6	-129.6	101.8	129
AH-17	275.6	12.19	163.3	92	ZJ-3	223.7	-72.93	97.28	130
AH-13	290.0	-9.734	162.3	93	AH-9	219.7	-69.45	96.49	131
HN-22	304.5	-30.27	161.8	94	ZJ-15	183.9	-31.33	92.17	132
AH-22	295.05	-20.95	160.4	95	ZJ-2	217.8	-80.60	90.63	133
HB-15	236.8	51.28	157.7	96	ZJ-16	176.3	-27.85	89.33	134
HB-30	217.1	73.38	155.9	97	AH-20	204.7	-66.77	89.05	135
ZJ-4	287.3	-25.04	154.2	98	AH-39	215.2	-87.06	86.40	136
AH-40	281.8	-19.06	153.6	99	AH-42	240.3	-126.1	84.17	137
HB-31	212.3	71.45	152.3	100	HB-7	178.8	-53.15	79.96	138
HN-17	266.8	-5.334	150.9	101	AH-3	192.6	-80.29	76.34	139
ZJ-45	254.2	6.544	148.7	102	AH-12	201.5	-100.2	72.96	140
AH-2	262.6	-12.41	145.4	103	AH-36	216.8	-129.6	69.18	141
HB-13	227.9	26.87	142.2	104	AH-34	189.6	-95.54	68.09	142
HB-34	191.6	75.09	142.0	105	HB-8	165.5	-65.05	67.27	143
ZJ-17	236.1	6.815	138.4	106	HB-12	203.1	-119.3	65.72	144
AH-32	261.6	-30.33	137.2	107	HB-11	198.9	-117.7	64.00	145
HB-32	183.2	66.12	133.3	108	AH-38	201.3	-133.4	58.65	146
AH-26	282.4	-67.86	133.2	109	HB-9	151.4	-72.77	55.91	147
ZJ-22	253.3	-29.45	132.8	110	AH-7	185.3	-120.1	55.16	148
HN-25	307.6	-106.4	131.2	111	HB-3	143.6	-86.17	45.70	149
HN-16	250.1	-29.29	131.0	112	ZJ-21	154.9	-126.5	35.03	150
HB-35	188.8	50.17	129.7	113	AH-31	160.1	-151.3	27.43	151
ZJ-9	254.9	-42.96	128.0	114	HB-10	157.1	-166.1	19.34	152

Table S5. The color parameter descriptive statistics of Kruskal-Wallis test

Project	Origins	ZJ	AH	HN	HB
	Reference ranges	64.37~79.10	71.08~83.96	52.79~77.11	60.49~83.20
	Mean	71.72	79.10	66.23	72.23
*L**	Mean ranks	62.71	117.0	37.78	73.54
	Kruskal-Wallis H	62.87			
	P value	< 0.0001			
	Reference ranges	1.57~6.70	1.34~4.68	3.14~7.40	0.96~6.16
	Mean	3.699	2.376	4.999	3.500
*a**	Mean ranks	85.25	43.77	119.2	73.20
	Kruskal-Wallis H	51.74			
	P value	< 0.0001			
	Reference ranges	17.07~27.12	13.60~23.24	20.60~25.19	13.27~27.14
	Mean	21.04	17.50	23.55	19.97
*b**	Mean ranks	85.38	41.68	121.6	73.77
	Kruskal-Wallis H	57.93			
	P value	< 0.0001			

Table S6. The results of physicochemical content prediction model based on Electronic-Nose

Algorithm	Physicochemical Index	R^2	MAE	MBE	RMSE
BPNN	Extracts	0.2963	4.8493	1.1531	5.9913
	Essential oil	0.3573	0.1612	0.0418	0.1888
	Polysaccharide content	0.0972	83.4825	-8.6937	116.3458
	Atractylenolide I	-0.0549	3.3783	-1.0090	4.1387
	Atractylenolide II	0.0006	3.6246	0.1479	4.7807
	Atractylenolide III	-0.2318	6.8464	-2.4376	11.5298
	Biatractylolide	-0.0194	2.7374	0.9297	3.5607
	Atractylone	0.0289	144.1505	6.4465	175.7386
PLS-DR	Extracts	0.2791	4.8771	0.9048	6.0640
	Essential oil	0.1796	0.1761	0.0966	0.2133
	Polysaccharide content	0.1635	83.2015	-2.0641	111.9910
	Atractylenolide I	0.1270	3.2002	0.1155	3.7650
	Atractylenolide II	0.1701	3.3699	-0.7059	4.3565
	Atractylenolide III	0.0069	6.7989	-1.1873	10.3523
	Biatractylolide	-0.0443	3.0059	0.8791	3.6040
	Atractylone	0.0561	138.9834	44.1382	173.2654
PSO-SVM	Extracts	0.4233	4.3638	1.0887	5.4238
	Essential oil	0.0526	0.1677	0.0308	0.2292
	Polysaccharide content	0.1707	77.6528	-11.0218	111.5093
	Atractylenolide I	0.0945	3.0914	-0.8128	3.8344
	Atractylenolide II	-0.0573	3.5511	-1.3402	4.9172
	Atractylenolide III	-0.1719	6.9911	-3.8652	11.2459
	Biatractylolide	-0.0861	2.8673	0.4667	3.6755
	Atractylone	0.1426	119.1989	25.0560	165.1323

*: MAE: Mean Absolute Error; MBE: Mean Bias Error; RMSE: Root Mean Square Error.

Table S7. The results of physicochemical content prediction model based on color parameters

Algorithm	Physicochemical Index	R^2	MAE	MBE	RMSE
BPNN	Extracts	0.45636	4.336	-0.049218	5.2659
	Essential oil	-0.0596	0.18897	0.033237	0.24236
	Polysaccharide content	0.46186	72.762	11.4207	89.8266
	Atractylenolide I	0.88244	0.43149	0.089938	0.51536
	Atractylenolide II	-0.01497	4.0891	0.38416	4.8178
	Atractylenolide III	-0.19252	7.9184	1.8794	11.3444
	Biatractylolide	0.1063	2.3928	0.44119	3.3341
	Atractylone	-0.18625	149.6579	-12.2285	194.2347
PLS-DR	Extracts	0.39649	4.7148	0.51785	5.5482
	Essential oil	-0.0795	0.21322	0.088828	0.24463
	Polysaccharide content	0.58849	63.892	0.12847	78.5501
	Atractylenolide I	0.91014	0.34453	0.012493	0.45056
	Atractylenolide II	0.079412	3.5902	0.01548	4.5883
	Atractylenolide III	0.067906	7.2878	0.0108	10.0295
	Biatractylolide	0.12445	2.431	0.7449	3.3
	Atractylone	0.043207	135.5219	23.917	174.4408
PSO-SVM	Extracts	0.33759	4.4147	-0.55155	5.8127
	Essential oil	-0.31303	0.19647	0.079327	0.2698
	Polysaccharide content	0.63968	54.614	1.9284	73.5024
	Atractylenolide I	0.9096	0.34599	-0.041259	0.45193
	Atractylenolide II	0.098122	3.3962	-1.2226	4.5415
	Atractylenolide III	0.14146	6.329	-3.2834	9.6257
	Biatractylolide	0.04621	2.5211	0.36584	3.4443
	Atractylone	-0.00264	131.0749	18.7147	178.5711

*: MAE: Mean Absolute Error; MBE: Mean Bias Error; RMSE: Root Mean Square Error.
